# Outpatient care for pregnant and puerperal women during the COVID-19 pandemic: Number 3 - September 2020

**DOI:** 10.1055/s-0040-1718473

**Published:** 2020-09-29

**Authors:** Fernanda Garanhani de Castro Surita, Adriana Gomes Luz, Lilian de Paiva Rodrigues Hsu, Francisco Herlânio Costa Carvalho, Marianna Facchinetti Brock, Mary Uchiyama Nakamura

**Affiliations:** 1 Departamento de Tocoginecologia, Faculdade de Ciêcias Médicas, Universidade Estadual de Campinas, Campinas, SP, Brazil; 2 Faculdade de Ciêcias Médicas da Santa Casa de São Paulo, São Paulo, SP, Brazil; 3 Faculdade de Medicina, Universidade Federal do Ceará, Fortaleza, CE, Brazil; 4 Universidade do Estado do Amazonas, Manaus, AM, Brazil; 5 Escola Paulista de Medicina, Universidade Federal de São Paulo, São Paulo, SP, Brazil

## Key-points

- The COVID-19 pandemic remains a rapidly evolving situation, and as new research and data become available, clinical care recommendations should be refined to reflect the most current information.
- Importance of maintaining outpatient care for pregnant and postpartum women during the pandemic period due to the new coronavirus
- Provide care so that consultations do not become a place of risk for contamination of users and health professionals. This care must involve the physical space, use of PPE, guidance from professionals and pregnant and postpartum women
- Importance of vaccination of pregnant women for influenza, thus facilitating the differential diagnosis of respiratory syndromes
- Provide strategies for health education in prenatal care and postpartum care using new technologies
- The more expanded the testing for COVID-19, the more it provides protection for professionals and patients

## Recommendations

- During the pandemic and, as long there is a risk of contamination by COVID-19, outpatient care for pregnant and puerperal women needs to be maintained, however additional strategies need to be implemented;
- Restructure health services in order to screen symptomatic individuals, provide adequate use of Personal Protective Equipment (PPEs) by health professionals;
- Promote social distancing without leaving aside the welcoming and humanization aspect of the care for pregnant and postpartum women;
- Think new ways to carry out groups of pregnant women minimizing risk of contamination, using educational videos, mobile phone applications, online groups;
- Whenever possible, universal screening promotes greater security for women, newborns and health professionals and should be done;

## Background

On March 11th, 2020, the World Health Organization (WHO) decreed a pandemic state for the coronavirus 19, named COVID-19. The rapid evolution of the outbreak, which spread across all continents, generated universal concern, not only for the number of deaths, but also for the worldwide impact in the most diverse spheres. 1 In order to prevent further spread of the epidemic, people are advised to stay at home, avoid crowds and promote social distancing, which poses a dilemma for many women during the pregnant puerperal cycle about the possibility of going to hospitals, doctors's offices, laboratories or primary health care units.

Prenatal care is known to be important throughout pregnancy, to identify risk, prevention and management of pregnancy-specific diseases or pre-existing pathological conditions, patient education and health promotion. 2

And the same risks and questions apply to outpatient postpartum consultations, a period in a woman's life that even under normal conditions is neglected. Currently, the postpartum period has been the focus of many new studies due to its importance in caring for women, not only in the immediate postpartum period, but also in the long term, considering reproductive planning, mental health, self-care and also in the follow-up guidance for comorbidities.

So far, there are disagreements about what is the risk for pregnant women,in terms of susceptibility or adverse results in SARS-CoV-2 infection (severe acute respiratory syndrome by coronavirus) compared to the general population. Some respiratory diseases, such as H1N1, have been associated with worse results in pregnant women, but initial studies on COVID-19 infection among pregnant women have not shown this increased risk. 3 4 However, we need to cite national data with a significant increase in maternal mortality from infection with the new coronavirus. 5

More recent data show a greater concern among affected pregnant women. Serious manifestations of the disease, admission to the ICU and mechanical ventilation were more frequent among pregnant women, although there is no description of increased mortality rates so far. 6 Adverse perinatal results such as increased rates of prematurity and fetal death have also been reported. 7 However, the mortality data should be revised and adjusted by age, and may bring new results in the future.

Pregnant women with chronic diseases (hypertension, diabetes) or obesity should be considered at higher risk for complications of COVID-19 infection as well as the general population. 5

However, it is known that prenatal and postnatal follow-up should not be suspended, given the importance of monitoring and following-up in decreasing maternal-fetal, neonatal and puerperal risks.

## How to structure the changes in outpatient care for pregnant and puerperal women while there is a risk of infection for COVID-19?

The risk of going to health units, doctor's offices or just leaving home should be considered at each scheduled prenatal visit. Alternative approaches in providing antenatal care have been proposed as a strategy in the effort to control the spread of COVID-19 among patients, caregivers and staff. Even if evidence is limited in relation to the safety and effectiveness of these approaches, several international entities recognize the need to implement innovative strategies during this rapidly evolving public health emergency, considering the differences in the care environments and the population's risks.

Thus, consultations should be used in the best possible way with the greatest amount of clarification and guidance that can be carried out. We must consider grouping the components of the treatment (for example, vaccines, blood glucose tests, ultrasound examinations, etc.) However, for some situations of high-risk pregnant women and in the third trimester, there is no way to guide waiving or spacing of prenatal visits. Frequent situations such as those of pregnant women with any form of hypertension, diabetes, fetal growth restriction cannot be left without qualified outpatient follow-up as it could increase the risk of perinatal morbidity and mortality. In addition to these, other conditions also cannot go without close monitoring, as social vulnerability and serious illnesses (heart disease, autoimmune diseases, neoplasms).

The burden of this unprecedented situation we are experiencing can be somehow minimized with the organization of health services that assist pregnant and postpartum women.

Since the beginning of the pandemic, numerous service protocols have been created and updated at a unique speed that makes it difficult to stay updated on the topic. However, some precautions are consensus both in the structuring of services and in individual care. It is recommended to review the existing flows for early identification and immediate care in a specific location for “COVID care” for symptomatic pregnant and postpartum women, optimizing care. And, in a general way for all pregnant and postpartum women to reduce their stay in Health Units. 8

## What changes in the structuring of services should be implemented?

- If possible, previously telephone contact the patient to ask about covid-19 signs and symptoms: fever, cough, runny nose, body pain, diarrhea, abdominal pain. If symptoms are present, advise seeking specific care for patients at risk of covid-19 and not going to the routine prenatal/postpartum outpatient service to avoid contact with other pregnant/postpartum women;
- Pre-service screening for every pregnant and puerperal woman arriving at outpatient clinic service with questions about the signs and symptoms described above and temperature measurement;
- To favor the social distancing between users of the health service, with the delimitation of the physical space used and the marking of armchairs / chairs in the waiting room. To avoid a greater number of people in the same place, during this period the routine presence of a companion should be avoided, which can be reviewed in special situations;
- And that health services have different entrances, physical space and work teams for the care of pregnant and puerperal women with and without symptoms and signs suggestive of SARS-Cov2.

Thus, it is essential to reorganize the health service to provide adequate care for pregnant and puerperal women.

## What changes in individual care should be made?

- The doctor should wash his hands before and at the end of the consultation, in the absence of a suitable place for hand washing, hand sanitizer with alcohol gel can be used. 5
- The use of medical masks (surgical and N95 / PPF2) is a preventive measure that limit the spread of respiratory diseases, including COVID-19. However, the use of a mask alone is not sufficient to provide the appropriate level of protection. Other equally relevant measures must be adopted. When using masks, this measure must be combined with hand hygiene and other preventive measures to prevent COVID-19 person-to-person transmission. Cloth masks, homemade, with common and low-cost materials should be used by pregnant women as an additional voluntary public health measure. 9
- Use of eye protection (Faceshield / protection glasses) by health professionals throughout outpatient care. This material must be reused after cleaning and disinfection. We emphasize that ordinary glasses for refractive corrections do not replace the recommended eye protection. 5
- Guide and reinforce the importance of influenza vaccination during prenatal care.
- Evaluate the possibility of extending the interval between consultations, as long as it does not compromise clinical and obstetric issues.
- Optimize collection of laboratory exams, ultrasounds and other subsidiary exams, so that they are carried out on the same days as the consultations, within the possibilities of the services, avoiding, whenever possible, leaving the home and excessive exposure of pregnant women and postpartum.
- Due to the impossibility of keeping the distance safe, there is a great risk of contamination during ultrasound (USG). Therefore, ultrasound examinations should be reduced to the essential minimum and patients should attend without companions. For the pandemic period, the following are recommended: USG in the first trimester between 11 and 13 weeks (to date pregnancy and the first trimester morphological exam); 18 to 24 weeks USG (for second trimester morphological assessment). The third trimester exam should only be performed if there is a clinical indication. In pregnancies with maternal or fetal pathologies, strict monitoring with the minimum necessary frequency is justified. In pregnant women with infection confirmed by COVID 19, ultrasound exams should be postponed as much as possible to reduce the spread of the virus. 10

## How to manage some medications used frequently in pregnancy?

Below are some guidelines on the use of some medications that may be needed during prenatal care and what changes if you suspect or confirm a COVID-19 infection:

- Betamethasone: - the Center for Disease Control and Prevention (CDC) initially recommended avoiding glucocorticoids in pregnant women positive for COVID-19, because an association with increased risk of mortality in influenza patients (coronavirus infection in MERS-CoV) has been shown. Due to the neonatal benefits of prenatal administration of betamethasone for fetal lung maturation between 24 + 0 and 33 + 6 weeks, when there is a risk of preterm birth, the American College of Obstetrics and Gynecology (ACOG) continues recommending its use for the standard indications for pregnant patients even those with suspected or confirmed for COVID-19. 11 However, these decisions can be individualized, always weighing the neonatal benefits with the risks of possible harm to the pregnant woman.
- Low dose aspirin - For pregnant women without COVID-19, ACOG advises maintaining the use of low dose aspirin as clinically indicated (for example, prevention of pre-eclampsia). For those with suspicion or confirmation of COVID-19 with indication of low-dose aspirin, the decision to continue the drug must be individualized, and it is usually possible. Given the lack of data, the European Medicines Agency and the World Health Organization do not recommend avoiding NSAIDs in patients with COVID-19 when clinically indicated. 12

## Asymptomatic obstetric patients and universal testing - What do we know?

In the first study in the USA testing COVID-19 in 100% of pregnant women (n = 215), 1.9% were symptomatic and tested positive for COVID-19, among asymptomatic 84.6% tested negative and 13.5 % tested positive for COVID-19. This study was in parturient women, but probably the proportion among pregnant women in prenatal care is similar. 13 Also in the USA, another study drew attention to the low prevalence of COVID-19 (2.7% [5/188]) in universal testing among pregnant women and puerperal women, and among asymptomatic, only 2 positive cases, which were negative in the 2nd sample. 14 However, in a study in Japan, the percentage of positive tests among asymptomatic obstetric patients was 4%. 15

So far, we do not have universal testing data in Brazil, but national studies are being carried out for this purpose. While we do not have all data, everyone, health professionals and pregnant/puerperal women must protect themselves and prevent the spread of the virus. 16

## How to innovate in antenatal education and mental health care?

Social distancing makes it impossible to carry out groups pregnant women. Antenatal education groups have been increasingly valued, due to the possibility of different orientations by a multidisciplinary team, not only about the evolution of pregnancy and childbirth. These groups deal with issues that are important for women and that are usually superficially addressed in individual medical appointments, due to lack of time or other difficulties. Rights of pregnant women, possibilities of contraception in the immediate postpartum or postpartum consultation, nutritional aspects, domestic violence, depression and anxiety, physical activity, breastfeeding and care for newborns are topics that need to be discussed during prenatal care , so that women feel more empowered and have a positive experience in their pregnancy and postpartum period. (2)

It is necessary to think of new ways to reinvent the activities carried out in groups for pregnant women, through the use of educational videos, mobile phone applications, online groups, in short, all types of educational / informational material that could be offered without risk of contamination. In addition to making the most of individual consultation time for these approaches.

Another differential of this pandemic period is the increase in anxiety, sadness and fear. The uncertain scenario related to the disease, pregnant and puerperal women infected or not with COVID-19 may be experiencing intense psychological suffering, which can cause serious consequences in terms of mental health. (17) It would be interesting for pregnant and puerperal women to have a way of communicate with health professionals in case of psychological distress or to resolve doubts during this period.

Studies have shown poor sleep quality in pregnant women and it is observed that sleep disorders seem to worsen throughout pregnancy, which may have impacts on labor, maternal and fetal health. Poor sleep quality can be even greater in these pandemic periods with social distancing, reduced leisure activities and anxiety. (18 19)

All these details in outpatient care must include women in pregnancy in the postpartum period. We cannot neglect women in the postpartum period, as it is known that most maternal deaths occur in the puerperium and are related to delay or difficulty in accessing health services after the woman's hospital discharge.

## Final considerations

The priority today is to reduce the public health burden of COVID-19. We obstetricians and gynecologists have an obligation to help implement simple measures to make pregnant women and postpartum women aware of safe practices such as hand hygiene, wearing masks, social distancing, cough etiquette, staying at home whenever possible and disinfecting surfaces often. For as long as there is no vaccine, these precautions are what can reduce contamination and save lives. We cannot refrain from offering qualified care focused on the needs of each woman, and try to provide, even in times of exception like this pandemic, a positive and safe experience during pregnancy, childbirth and the puerperium as recommended by the WHO good practices.

National Specialized Commission on Prenatal Care of the Brazilian Federation of Gynecology and Obstetrics Associations (FEBRASGO)

President:

Fernanda Garanhani de Castro Surita

Vice-President:

Lilian de Paiva Rodrigues Hsu

Secretary:

Adriana Gomes Luz

Members:

Edson Gomes Tristão

Eliana Martorano Amaral

Eugenia Glaucy Moura Ferreira

Francisco Herlanio Costa Carvalho

Joeline Maria Cleto Cerqueira

José Meirelles Filho

Luciana Silva dos Anjos França

Marianna Facchinetti Brock

Mary Uchiyama Nakamura

Patricia Gonçalves Teixeira

Renato Ajeje

Sérgio Hecker Luz
